# Representation of Afferent Signals from Forearm Muscle and Cutaneous Nerves in the Primary Somatosensory Cortex of the Macaque Monkey

**DOI:** 10.1371/journal.pone.0163948

**Published:** 2016-10-04

**Authors:** Hiroshi Yamada, Hiroaki Yaguchi, Saeka Tomatsu, Tomohiko Takei, Tomomichi Oya, Kazuhiko Seki

**Affiliations:** 1 Department of Neurophysiology, National Institute of Neuroscience, National Center of Neurology and Psychiatry, Kodaira, Tokyo, 187-8502, Japan; 2 Division of Biomedical Science, Faculty of Medicine, University of Tsukuba, Tsukuba, Ibaraki, 305-8577, Japan; 3 Graduate School of Comprehensive Human Sciences, University of Tsukuba, Tsukuba, Ibaraki, 305-8577, Japan; 4 Precursory Research for Embryonic Science and Technology (PRESTO), Japan Science and Technology Agency (JST), Saitama, 332-0012, Japan; University of Chicago, UNITED STATES

## Abstract

Proprioception is one’s overall sense of the relative positions and movements of the various parts of one’s body. The primary somatosensory cortex (SI) is involved in generating the proprioception by receiving peripheral sensory inputs from both cutaneous and muscle afferents. In particular, area 3a receives input from muscle afferents and areas 3b and 1 from cutaneous afferents. However, segregation of two sensory inputs to these cortical areas has not been evaluated quantitatively because of methodological difficulties in distinguishing the incoming signals. To overcome this, we applied electrical stimulation separately to two forearm nerves innervating muscle (deep radial nerve) and skin (superficial radial nerve), and examined the spatiotemporal distribution of sensory evoked potentials (SEPs) in SI of anaesthetized macaques. The SEPs arising from the deep radial nerve were observed exclusively at the bottom of central sulcus (CS), which was identified as area 3a using histological reconstruction. In contrast, SEPs evoked by stimulation of the superficial radial nerve were observed in the superficial part of SI, identified as areas 3b and 1. In addition to these earlier, larger potentials, we also found small and slightly delayed SEPs evoked by cutaneous nerve stimulation in area 3a. Coexistence of the SEPs from both deep and superficial radial nerves suggests that area 3a could integrate muscle and cutaneous signals to shape proprioception.

## Introduction

Proprioceptive signals shape perception of the relative positions and movements of the various parts of the body. Self-movements activate peripheral sensory receptors that signal the current state of the limb and body to the cerebral cortex [1, 2]. These proprioceptive signals are indispensable for constructing precise and complex limb and body movements. Disruption of this sensorimotor link impairs movements, even when the motor system remains intact. For example, sensory deafferentation results in inaccurate hand movements in human patients [3, 4]. However, how the proprioceptive signals are processed in the primary somatosensory cortex (SI) remains unclear.

Area 3a, a part of SI located at the bottom of CS, responds to proprioceptive input [5] and sends projections to the primary motor cortex (MI), presumably to adjust motor output based on sensory feedback [6–12]. Lesions in area 3a cause changes in the proprioceptive signals represented in the secondary somatosensory cortex (SII) [13] that may result in motor deficits [14]. Inputs and outputs from these areas around CS have been examined extensively in anatomical tracing studies [15–20]. These studies suggest that proprioceptive signals are conveyed to area 3a. Moreover, these proprioceptive signals go through the cuneate nucleus and thalamus and are distinct from tactile signals conveyed to the rest of SI. These proprioceptive and tactile signals have been suggested to converge in area 2, but not elsewhere in SI [21].

A small number of electrophysiological studies in awake animals have suggested that area 3a neurons represent positions of corresponding body parts during active and passive movements [22–25]. In most of these studies, however, it has been extremely difficult to dissociate proprioceptive signals from tactile signals because traditional stimulation protocols do not dissociate the proprioceptive and tactile aspects of the stimulus. A recent study [26] attempted to address this issue by developing a mechanical stimulator that reliably positioned monkey’s hands in different postures to induce different proprioceptive states while also having precise control over tactile stimulation to the hand. This method appeared to successfully disentangle contribution of the proprioceptive and tactile inputs to neuronal activity in SI. However, it is known that different body postures modulate activities of both superficial (e.g., stretching the surface of skin) and deep receptors (e.g., stretching muscle spindles), and thus, it remains unknown how these two afferent signals induce neuronal activity in area 3a to indicate distinct proprioceptive states. Indeed, proprioception is known to be generated from deep [27, 28] and superficial receptors [29–33]. Therefore, it is important to identify whether and how the somatosensory signals derived from these two peripheral sensory receptors are represented in area 3a.

In this study, we used a method that overcomes the difficulty mentioned above: a receptor-specific identification of cortical areas 3a and 3b using afferent electrical stimulation on implantable nerve cuff electrodes. We analyzed spatiotemporal distribution of the SEPs in SI using electrical stimulation of afferents innervating muscle-joint (deep radial nerve, DR) or cutaneous (superficial radial nerve, SR) receptors in the dorsal (extensor) aspect of the forearm [34]. Using anaesthetized macaque monkeys, we addressed the following questions: i) do inputs from muscle-joint and cutaneous afferents converge in area 3a and adjoining regions of SI?; and ii) to what extent do these different modalities converge in SI? Our results suggest that area 3a may not only represent the signals from muscle-joint afferents, but may also integrate them with the cutaneous signals, a role typically reserved for cortical areas that are further downstream.

## Materials and Methods

We used two rhesus monkeys (Macaca mulatta; monkey SO, 10 kg, male; monkey TA, 7 kg, male). Experiments took place at the Department of Neurophysiology, National Institute of Neuroscience. Each animal was individually housed in a cage (80 × 70 × 80 cm) in the primate research facility of the National Institute of Neuroscience. Several cages were stationed in the same room, permitting interaction between monkeys. The room was maintained under a 12/12-h light/dark cycle. Water was available ad libitum and a standard commercially formulated non-human primate diet (AS; Oriental Yeast, Tokyo, Japan) was provided once daily (120 g/meal) and supplemented daily with a variety of vegetables, fruits, and grains. Toys were given to each monkey as part of environmental enrichment protocol. Regular care and monitoring, balanced nutrition, and environmental enrichment were provided by staffs of the center. Each monkey’s health and weight were routinely monitored by an expert veterinarian who was available on site. Animals were sacrificed at the end of the study with an intravenous injection of 1 mL of heparin after an overdose of pentobarbital injection (50 mg/kg), followed by transcardial perfusion.

All experimental procedures were approved by the Primate Research Committee at the National Institute of Neuroscience, NCNP, Japan and were performed in accordance with the Guide for the Care and Use of Laboratory Animals (National Research Council of the National Academies in the USA). Anaesthesia was induced with ketamine hydrochloride (5 mg/kg), xylazine (1 mg/kg), and buprenorphine (10 μg/kg), then maintained with propofol (12 mg/kg/h) and additional buprenorphine (10 μg/kg/6h). Heart rate, blood pressure, percutaneous oxygen saturation, and end-tidal CO2 were monitored and maintained throughout anesthesia. Durations of the experiments were 32 and 30 h in monkeys SO and TA, respectively.

### Nerve stimulation

**Fig 1:**
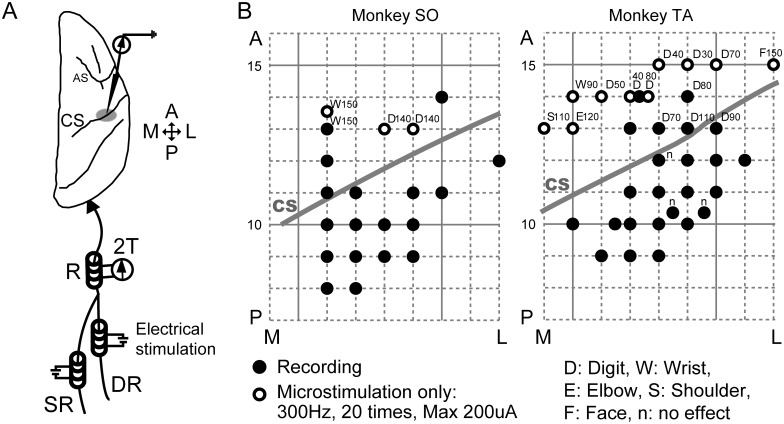
Schematic drawing of experimental setting and surface map of the recording sites. (A) Stimulation of deep radial (DR) and superficial radial (SR) nerves. Three nerve cuffs were implanted: one on the radial nerve trunk (R) at the left forearm, one on the DR, representing primarily muscle afferent input, and one on the SR, representing primarily input from the skin. The DR and SR cuffs were used for electrical stimulation, and the R cuff was used for recording incoming volleys. The nerves were stimulated with biphasic constant-current pulses, 100 μs/phase, at twice the threshold (2T). The electrical stimulation-evoked field potential was recorded from the forearm region at the posterior bank of the CS of the right hemisphere. (B) Cortical surface map of the recording sites in each monkey. The electrode was inserted 8–15 mm at the anterior–posterior level. Gray lines indicate the approximate location of the CS on the cortical surface. Recording sites of the SR- or DR-evoked potentials are indicated by filled circles. Open circles indicate electrode insertions in which intracortical microstimulations were applied and no SEPs were recorded. Body parts activated at the lowest current of the microstimulation are indicated by capital letters. Values indicate the lowest microstimulation current (μA) evoking the movement. “n” indicates no effect up to 200 μA. A: anterior, P: posterior, M: medial, L: lateral.

Three nerve cuffs were implanted: one on the radial nerve trunk (R) at the forearm; one on the deep radial nerve (DR), representing afferent input from muscle spindles, tendon organs, and joint receptors; and one on the superficial radial nerve (SR), representing input from the skin (Fig 1A). After implanting the electrodes, the incision was closed with sutures. DR and SR cuffs were used to apply electrical stimuli to each nerve, and R cuffs were used to record incoming volleys evoked by the stimuli. Each nerve was stimulated with biphasic constant-current pulses with a duration of 100 μs. Intensity of the electrical stimuli was set at twice the threshold (2T) that elicited barely detectable volleys recorded in the R cuff. At the beginning of the recording session, thresholds for DR and SR were set at 250 and 300 μA in monkey SO, and 260 and 350 μA in monkey TA, respectively. Throughout the experiment, the threshold current was monitored and remeasured if the volley amplitude from the R cuff electrode changed.

### Data recording and analysis

A craniotomy exposed frontal and parietal cortices. The dura mater was removed and the tissue surface covered with paraffin oil throughout the recording. A glass-coated tungsten microelectrode (0.5–1.0 MΩ) was inserted into the anterior and posterior parts of CS. Regions of SI with receptive fields in the forearm and hand were targeted and located with reference to the arcuate sulcus. The electrode was advanced through gray matter until it reached white matter by determining whether no unit activity and low background noise level were detected. The SEPs induced by the electrical stimuli to DR and SR (single biphasic constant-current pulse, 2.22 Hz, 100 μs/phase, *n* = 60 each) were recorded together with the incoming volleys from the R cuff electrode at a sampling rate of 10 kHz with a band-pass filter at 100 Hz to 20 kHz. SEPs for each penetration were recorded at points of varying depth, with 1 or 2 mm between the consecutive depths. Recording sites spanned from the surface to bottom of the posterior part of CS.

**Fig 2:**
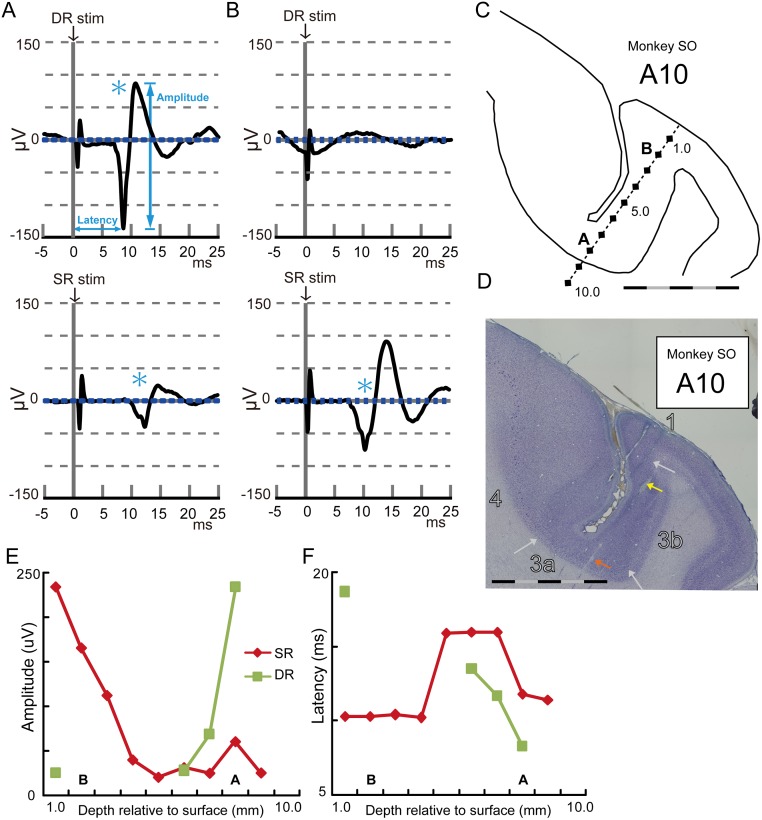
Representative examples of DR- and SR-evoked potentials. (A) An example of the DR- and SR-evoked potentials recorded at the bottom of the posterior part of CS. Averaged traces of 60 trials are shown at the time aligned with DR and SR stimulations. An asterisk indicates a detected response compared with the baseline activity. Blue dotted lines indicate one standard deviation from the baseline activity. (B) Same as (A) except for recording at the superficial part of the posterior part of CS. (C) Reconstructed trace of the electrode insertion in which the SEPs in A and B were recorded. (D) An example of the thionine-stained coronal section in monkey SO. White arrows indicate the approximate locations of the borders of areas 4, 3a, 3b, and 1. Colored arrows indicate the lesion marks. The scale bars in C and D indicate 5 mm, separated by black and gray every 1 mm. (E) Amplitude plots of the DR- and SR-evoked potentials. The potentials were recorded every 1 mm from the surface to the bottom of the posterior part of CS as shown in C. (F) Latency plots of the DR- and SR-evoked potentials. In C, E, and F, characters A and B indicate the recording depth of the SEPs represented in A and B, respectively.

The SEPs were aligned with the onset of nerve stimulation and averaged (*n* = 60) at each recording site. We defined baseline activity as activity in the epoch ranging from 10 to 5 ms (5 ms duration) prior to SR stimulation. A significant response (Fig 2A, asterisks) was identified if the activity changes were above or below three standard deviations (SDs) of the baseline activity and if both first negative peak and following positive peak lasted more than 1 ms. Response latency was defined as the time from the onset of nerve stimulation to the first negative peak. Response amplitude was defined as the difference in potential between the first negative peak and the following positive peak.

The SEP latency and amplitude were plotted on electrode tracks reconstructed histologically and compared using ANOVA or two-sample *t*-tests, with statistical significance set at P < 0.05. Proportions of the recording sites with significant responses among subdivisions of SI were compared using Fisher’s exact probability test at P < 0.05. Note that locations of the recorded SEPs were identified and analyzed in each anterior-posterior level. To visualize all SEPs into one figure, all SEPs from multiple anterior-posterior level were overplayed into one typical histology section in each monkey.

### Intracortical stimulation

Electrical stimulation was applied to the anterior part of CS in the forearm-hand representation in MI, which was located with reference to the arcuate sulcus. A train consisting of 30 biphasic constant-current pulses (100 μs/phase) was applied at 333 Hz at 10–200 μA. These stimulus trains were applied at varying depths along a single penetration, with 1 or 2 mm spacing between consecutive stimulus sites. Electrical stimulation-evoked movement was visually monitored by two experimenters, and the body part activated at the lowest current of microstimulation was detected and recorded to construct a surface map in MI.

### Histology

Two small electrolytic lesions were made at the bottom and superficial parts of the posterior part of CS by applying direct anodal current (20 μA) for 30 s through tungsten microelectrodes. The monkeys were deeply anaesthetized with pentobarbital (50 mg/kg, i.v.) and perfused transcardially with 10% formalin in 0.9% NaCl solution. Coronal sections of the right hemisphere 50 μm in thickness were stained with thionine. Stained segments and electrode tracks were reconstructed on the histology sections using the electrolytic lesion marks as reference points, and the recording sites were identified.

### Identification of Brodmann’s areas 4, 3a, 3b, and 1 and their borders

All procedures for identifications of the subdivisions at the anterior and posterior parts of CS were performed in accordance with Jones *et al*. (1978). We identified cortical areas 4, 3a, 3b, and 1 and their approximate borders (indicated with white arrows in Fig 2D). As suggested by Jones *et al*. (1978), perfect delineation of area boundaries based solely on cytoarchitecture is difficult. The most distinctive delineation was between 3b and 1, whereas other boundaries were not clearly distinctive.

The border between areas 4 and 3a in the anterior bank of CS was defined as size and number of the giant, deeply stained pyramidal cells of layer V that declined gradually from area 4 to 3a. We traced location of the giant pyramidal cells for histological reconstruction and border detection. A continuous line of progressively smaller, giant cells can be usually seen descending towards the sulcus floor. At their border, the end of the thin, dysgranular layer IV of area 3a may occasionally coincide with the giant cells of area 4, although these features can overlap considerably. The border between areas 3a and 3b was based primarily on two features: the thickest gray matter in the posterior bank of CS with dense layers II to IV and a relatively cell-free layer V. At low magnification, each of these strata undergoes a progressive narrowing and becomes less distinct. The border between areas 3b and 1 can be identified from the area 1 feature that is more highly laminated than that in area 3b.

## Results

### Database

We performed 55 microelectrode insertions around CS of two monkeys (Fig 1B; 21 and 34 in monkeys SO and TA, respectively). Receptive fields of recording sites in SI were roughly estimated to be the parts of body that moved in response to stimulation of MI directly. SEPs derived from DR and SR were recorded in the SI recording sites if stimulation of the corresponding MI sites resulted in hand or arm movements. SEPs were recorded from 179 and 168 sites through 18 and 24 microelectrode insertions in monkeys SO and TA, respectively.

### Examples of muscle-joint (DR) and cutaneous (SR) nerve-evoked potentials

Representative examples of DR- and SR-evoked potentials in a single insertion of the electrode are shown in Fig 2. A large-amplitude and short-latency response was observed after DR stimulation (Fig 2A, top) at the bottom of posterior part of CS (Fig 2C, 8 mm deep from the surface). Latency of the first negative peak was 8.3 ms, with an amplitude of 234.5 μV. SR stimulation also induced a response at this recording site, but the evoked potential was delayed with a smaller amplitude compared with the DR-evoked potential (Fig 2A, bottom; latency = 11.8 ms, amplitude = 60.3 μV). In contrast, a large-amplitude and short-latency response *via* SR stimulation was observed (Fig 2B; bottom, latency = 10.3 ms, amplitude = 165.4 μV) at the surface of the posterior part of CS (Fig 2C; 2 mm deep from the surface). No significant response to DR stimulation was observed at this recording site (Fig 2B, top). A typical insertion, spanning from the surface to bottom of the posterior part of CS (Fig 2C), was identified on the histology sections by referring to the electrolytic lesion marks as reference points (Fig 2D; colored arrows). Through this insertion of the electrode, large-amplitude and short-latency responses to DR stimulation were observed at the bottom (depth 6–8 mm, Fig 2E, green). In contrast, SR-evoked potentials were observed from the surface to bottom with the amplitude decreasing as a function of electrode depth, but it never disappeared (Fig 2E, red). At the midpoint of penetration (i.e., 5.0–7.0 mm from the surface), the latency of SR-evoked potentials became longer (Fig 2F).

### Contrasting properties of DR- and SR-evoked potentials among areas 3a, 3b, and 1

**Fig 3:**
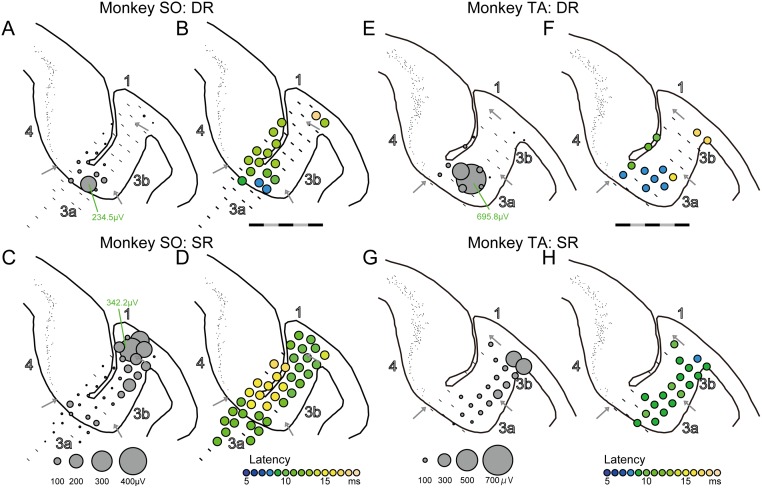
Examples of DR- and SR-evoked potential distribution in area 3a. (A) Amplitude plots of the DR-evoked potentials at the anterior-posterior 10-mm level in monkey SO where the largest DR-evoked potential was observed in this animal. The circle size indicates the amplitude. A bar indicates no significant activation. (B) Latency plots of the DR-evoked potential indicated by colors. (C, D) same as (A, B), but for the SR-evoked potentials. (E–H) Same as A-D but for monkey TA. The data in A11 were from where the largest DR-evoked potential was observed in this monkey. The scale bar indicates 5 mm, separated by black and gray every 1 mm.

We systematically examined whether these response properties were consistently observed in adjacent recording sites and whether they were related to the subdivision of SI defined by the cytoarchitecture. We first analyzed DR-evoked potentials at the anterior-posterior 10-mm level in monkey SO where the largest DR-evoked potential was observed in this animal (Fig 3A). Significant responses were clustered at the bottom in area 3a (88.9%, 8/9 sites). In contrast, fewer responses to DR stimulation were observed in areas 3b and 1 (3/17 sites, 17.6% in area 3b/1, Fisher’s exact probability test, P < 0.001). Latency of the response observed in area 3a was short (Fig 3B, mean ± S.E., 11.0 ± 2.1 ms), but it became longer as a function of distance from the most responsive cluster in area 3a (light green plots in area 3b or 4, 15.2 ± 3.1 ms in area 3b/1, two-sample *t*-test, P = 0.028). In the white matter close to area 3a at the bottom of CS (within 2.0 mm from the area 3a edge), no response was observed (0/8 sites, 0% in the white matter, Fisher’s exact probability test, P < 0.001). In contrast, SR-evoked potentials were observed even in area 3a (Fig 3C, 8/9 sites, 88.9% in area 3a, 17/17 sites, 100% in area 3b/1, P = 0.346), whereas the large-amplitude and short-latency responses were observed in areas 3b and 1 (Fig 3C and 3D). In the white matter close to area 3a at the bottom of CS (within 2.0 mm), small responses evoked by SR stimulation were frequently observed (7/8 sites, 87.5% in the white matter, Fisher’s exact probability test, P > 0.999). Thus, DR-evoked potentials were found in area 3a. In contrast, SR-evoked potentials were widely observed throughout SI.

Similar responses were also observed in monkey TA (Fig 3E–3H). We analyzed DR-evoked potentials at the anterior-posterior 11-mm level in monkey TA where the largest DR-evoked potential was observed in this animal (Fig 3E). DR-evoked potentials were observed more frequently in area 3a (Fig 3E, 8/12 sites, 66.7% in area 3a) than in areas 3b and 1 (2/11 sites, 18.2% in area 3b/1, Fisher’s exact probability test, P = 0.036). Latencies of the DR-evoked potentials observed in area 3a were short (Fig 3F, mean ± S.E., 8.8 ± 1.1 ms) compared with those in area 3b/1 (mean ± S.E., 17.5 ± 0.4 ms, two-sample *t*-test, P < 0.001). SR-evoked potentials were observed in both areas 3a (8/12 sites, 66.7%) and 3b/1 (10/11 sites, 90.9%, Fisher’s exact probability test, P = 0.317), although large potentials were observed in areas 3b and 1 (Fig 3G and 3H).

**Fig 4:**
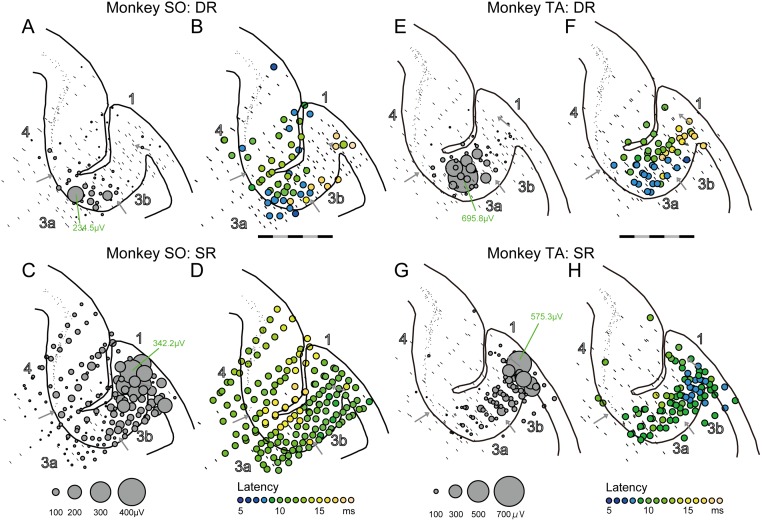
Overlaying plots of the DR- and SR-evoked potentials for all recording sites. (A) Amplitude of the DR-evoked potentials for all recording sites in monkey SO. The circle size indicates the amplitude. A bar at each recording site indicates no significant response. (B) Latency plots of the DR-evoked potentials for all recording sites in monkey SO. The latency is indicated by colors. The scale bar indicates 5 mm, separated by black and gray every 1 mm. (C, D) Same as (A, B), but for the SR-evoked potentials. (E–H) Same as A–D, but for monkey TA. All SEPs from multiple anterior-posterior level were overplayed into one typical histology section in each monkey.

**Fig 5:**
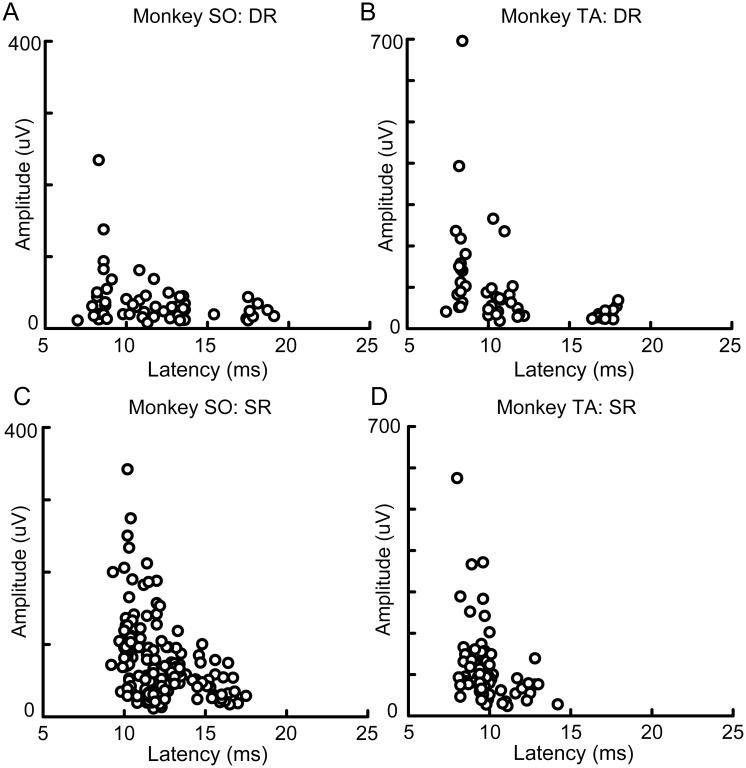
Relationship between response latency and amplitude of the DR- and SR-evoked potentials. (A) Plots of the DR-evoked potentials against latency and amplitude. The 57 responses observed from 179 recording sites in monkey SO are shown. (B) Same as A, but for monkey TA. The 50 responses of 168 recording sites are shown. (C, D) Same as (A, B), but for the SR-evoked potentials. The 163 responses observed from 179 recording sites in monkey SO (C) and 87 responses observed from 168 recording sites in monkey TA (D) are shown.

Next, we assessed whether these contrasting properties of the DR- and SR-evoked potentials were consistently observed at all recording sites in the posterior part of CS. We analyzed all SEPs of the reconstructed insertions in each monkey (Fig 4). As shown in overlaying plots of all SEPs from multiple anterior-posterior level, large DR-evoked potentials were observed in area 3a in both monkeys SO (Fig 4A) and TA (Fig 4E). Most of the large responses appeared clustered in area 3a at the bottom of the posterior part of CS in monkey SO and in area 3a close to the border of area 3b in monkey TA. Moreover, these large responses occurred at short latencies (Fig 4B and 4F) as the bluish plots in latency also clustered in the same location of the cluster in amplitude plots. A correlation analysis of all DR-evoked potentials revealed that large DR-evoked potentials occurred at a short latency (Fig 5A and 5B, correlation coefficient; r = -0.275, P = 0.038 in monkey SO, r = -0.405, P = 0.003 in monkey TA). This correlation was not strong because some short latency responses did not always appear with large amplitude (see bluish plots in MI; Fig 4A and 4B). These analyses indicated that DR-evoked potentials were evident exclusively in an area of 3a in each monkey, suggesting this area receives dense and strong inputs from the DR afferent that originates from proprioceptive receptors residing around hand and wrist extensor muscles.

In contrast to the DR-evoked potentials, SR-evoked potentials were widely observed at all recording sites in the posterior part of CS (Fig 4). The SR-evoked potentials were more frequently observed than the DR-evoked potentials (Fisher’s exact probability test; monkey SO, 163/179 sites, 91.1% *vs*. 57/179 sites, 31.8%, P < 0.001; monkey TA, 87/168 sites, 51.8% *vs*. 50/168 sites, 29.8%, P < 0.001), while most of the large responses were observed at the surface of the posterior part of CS in each monkey (Fig 4C and 4G). Consistent with the DR-evoked potentials, large SR-evoked potentials occurred with short latencies as the bluish plots in latency clustered at the surface of the posterior part of CS, especially in monkey TA (Fig 4H). A correlation analysis of all SR-evoked potentials revealed that large SR-evoked potentials occurred at a short latency in each monkey (Fig 5C and 5D, correlation coefficient; r = −0.388, P < 0.001 in monkey SO; r = −0.332, P = 0.002 in monkey TA). Again, the correlation was not strong because some short latency responses did not always appear with large amplitudes. Therefore, areas 3b and 1 were obviously the loci where the large-amplitude and short-latency SR-evoked potentials were observed. Indeed, neurons in some of these areas were activated by soft brushing stimulation to the skin surface of hand dorsum (online inspection). In addition, small SR-evoked potentials were observed in area 3a and over the posterior part of CS. Even in the anterior bank of CS, SR-evoked potentials were observed in monkey SO (Fig 4C and 4D). These results indicated that, although SR-evoked potentials were widely distributed in the posterior part of CS, large responses were predominantly localized in parts of areas 3b and 1 in each monkey.

### Small SR-evoked potentials in area 3a

**Fig 6:**
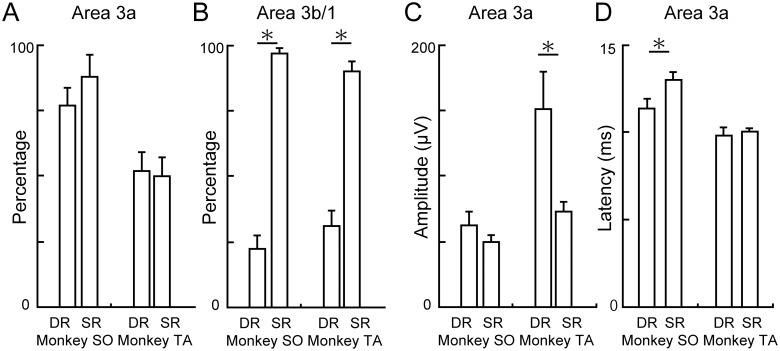
Characteristics of the SR-evoked potentials in area 3a. (A) Percent responses evoked by DR and SR stimulations in area 3a. (B) Percent responses evoked by DR and SR stimulations in area 3b/1. (C) Amplitude of the DR- and SR-evoked potentials in area 3a. (D) Latency of the DR- and SR-evoked potentials in area 3a. Error bars in A–D indicated S.E. Asterisk in A–D indicates statistical significance at *P* < 0.05 using two-sample *t*-test.

Small SR-evoked potentials (Fig 2A, bottom) were frequently observed in area 3a, as shown in Fig 4C and 4G. Next, we examined the SR-evoked potentials in area 3a quantitatively in comparison with the DR-evoked potentials (Fig 6). Proportions of the recording sites where a significant response was observed did not differ between SR and DR stimulation in area 3a (Fig 6A, Fisher’s exact probability test; monkey SO, 20/26 sites *vs*. 23/26 sites, P = 0.465; monkey TA, 25/48 sites *vs*. 24/48 sites, P > 0.999). This was in contrast to the DR-evoked potentials that were rarely observed in area 3b (Fig 6B, Fisher’s exact probability test; monkey SO, 15/67 sites *vs*. 65/67 sites, P < 0.001; monkey TA, 19/61 sites *vs*. 55/61 sites, P < 0.001). Therefore, SR- and DR-evoked potentials in area 3a were observed with similar frequencies. The amplitudes of the SR-evoked potentials observed in area 3a were smaller than the DR-evoked potentials, especially in monkey TA (Fig 6C, two-way ANOVA, nerve × monkey; nerve, P = 0.082, monkey, P = 0.001, interaction, P = 0.05). In monkey SO, they were not significantly different (two-sample *t*-test, P = 0.278) because the large-amplitude DR-evoked potentials were observed more focally, and hence, the averaged amplitude of the DR-evoked potentials observed all over area 3a was not large (Fig 6C, mean ± S.E., 62.3 ± 11.1 μV). However, it was clear that the SR-evoked potentials observed in area 3a were smaller than the large-amplitude DR-evoked potentials (top 10, mean ± S.E., 102.1 ± 21.0 μV, two-sample *t*-test, P = 0.012). Latency of the SR-evoked potentials observed in area 3a was longer than that of the DR-evoked potentials, especially in monkey SO (Fig 6D, two-way ANOVA, nerve × monkey; nerve, P = 0.035, monkey, P < 0.001, interaction, P = 0.125). In monkey TA, latency of the SR-evoked potentials observed in area 3a was longer than that of the large-amplitude DR-evoked potentials (top ten, mean and S.E., 8.8 ± 0.4 ms, two-sample *t*-test, P = 0.002). These results suggested that the small SR-evoked potentials could be elicited in area 3a, slightly delayed from the DR-evoked potentials.

In summary, large-amplitude and clustered DR-evoked potentials were observed in a ~2–3-mm cubic area in area 3a and its border to area 3b and 4, whereas the large SR-evoked potentials were observed only in area 3b/1 and were not observed in the area where the large DR-evoked potentials were observed. The small and slightly delayed SR-evoked potentials in area 3a were overlapped with the DR-evoked potentials.

## Discussion

In the present study we analyzed individual SEPs in the posterior part of CS of two anaesthetized macaques evoked by stimulating two forearm nerves innervating deep tissues and skin, DR and SR. The DR-evoked potentials were observed at the bottom of the posterior bank of CS, which corresponded to Brodmann’s area 3a. In contrast, the large SR-evoked potentials were observed at a short latency around the surface of the posterior part of CS, areas 3b and 1. Their amplitudes gradually decreased along the perpendicular axis to the surface, but they were still detectable in area 3a, although with a slightly delayed latency. Our results of receptor-specific characterization of SI suggest that neurons in area 3a carry proprioceptive signals, and further, they may also integrate them with cutaneous signals.

### Receptor-based characterization of cortical neurons

Isolation of proprioceptive and tactile stimuli to the hand of an awake monkey has been achieved recently by the use of a stimulator [26]. Using this novel device, Kim et al reported that single neurons in each area of SI, areas 3a and 3b, responded to both proprioceptive and cutaneous inputs. There are several significant differences between their findings and ours. First, we found overlapping representation of muscle and cutaneous signals in area 3a using local field potentials (LPFs), not by spiking activity of a single neuron. Because the LFPs reflect currents emerging from multiple sources [35], including synaptic inputs to the recorded area, it seems likely that the thalamocortical inputs to area 3a could be multimodal. Second and more importantly, receptor-based representation of sensory signals was examined in our study, while modality-based representation was examined in theirs. We implanted nerve cuff electrodes to monitor two afferent nerves from the muscle or skin of the forearm. This technique allowed us to successfully characterize the muscle and cutaneous afferent signals in the cortex separately. Receptor-based identification of sensory signals cannot be achieved with the device developed by Kim et al. because different body postures modulate the activities of both superficial (e.g., stretching the surface of skin) and deep receptors (e.g., stretching muscle spindle). In this study, we found that area 3a could represent the muscle and cutaneous afferent signals (Fig 6A), suggesting that area 3a may represent proprioception.

### Cutaneous representation in area 3a

Several reports have shown that some neurons in area 3a receive sub-threshold inputs from cutaneous nerves in cats and monkeys [36–39]. In the present study, we confirmed a similar overlapping representation of DR and SR inputs based on LFPs in area 3a of macaque monkeys (Fig 6A). The SR-evoked potentials in area 3a were small and slightly delayed versus the DR-evoked ones (Fig 6C and 6D). We did not characterize a functional relevance of this overlapping representation in the current study, but we suggest the following functions. One possibility is that the cutaneous representation in area 3a may largely reflect the fact that area 3a receives some tactile signals from the ventral lateral nucleus of the thalamus [19, 40]. In this case, overlapping representation of SR- and DR-evoked potentials within area 3a might indicate that proprioception (from DR afferent) and tactile (from SR afferent) signals are integrated within area 3a. Classically, this integration has been thought to occur in the postsynaptic area to area 3a [21]. For example, neurons in area 1 receive direct projection from both areas 3a and 3b [41], and consequently the integration of tactile and proprioception could occur at area 1 or a later stage. Recent reports, however, suggested that integration of higher-order peripheral information, like a haptic input feature [42] or edge-orientation [43], could occur subcortically via primary afferents. Thus, multimodal integration may also occur in earlier stages of cortical processing, as in area 3a.

Another possibility is that cutaneous representation in area 3a may largely reflect the fact that “proprioceptive component” of SR input was represented in area 3a, while “tactile component” was represented in area 3b. It is known that specific cutaneous afferents signal proprioceptive as well as tactile information [44, 45]. If this is the case, integration may occur in area 3a across the muscle and cutaneous afferent components of proprioception. The subsequent multimodal integration has been thought to occur at a late stage, such as area 1[6, 41]. Further study to characterize neurons in areas 3a and 3b may show the dominance between these two possibilities, perhaps using both a sophisticated mechanical stimulator [26] and receptor-specific nerve cuff stimulation.

In addition, it is also possible that the electrical stimulation to SR nerve might recruit myelinated afferent from A-fibre nociceptors [46, 47], and that may affects on the widespread representation of cutaneous input in the primary sensory cortex including area 3a.

### The cutaneous component of proprioception and its neural processing

It was first demonstrated by Edin & Johansson [33] that human proprioception is greatly affected by the pattern of strain applied over the skin surface independently from deep receptors. Several studies confirmed this phenomenon [32, 48–50], but their mechanism in the central nervous system is still unclear. MI in human subjects was involved in somatic perceptions of hand movements under movements illusion which were triggered by vibratory stimuli applied to forearm [51]. Since vibratory stimulation inevitably activate cutaneous receptors in addition to the targeted muscle-tendons, neurons in area 3a could be activated by both of these inputs, and proprioception could be shaped within area 3a or its upstream. Erroneous proprioceptive information should be represented in area 4 during the movement illusion. Therefore, proprioception could be attributed in part to the activity of area 3a and their projection to area 4 [15].

In this paper, we propose a receptor-based classification of cortical regions. This has an advantage of providing online identification of area 3a, which receives cutaneous inputs. Therefore, this method should contribute to determining the involvement of cutaneous signals in processing proprioception in the cortex, as in areas 3a and 4.

### SEP properties in anesthetized and behaving primates

SEPs from the muscle-joint and cutaneous nerves of the forearm have been qualitatively examined in anesthetized baboons in areas 3a and 3b [34, 39]. In these studies, the SEP properties, such as latency and amplitude, were qualitatively described in a manner consistent with our data in macaque monkeys. There are some inter-species differences in cortical structures of macaques and baboons [52, 53]. Together with the fact that functional roles of sensory cortices have been examined within a class of non-human primates primarily among behaving macaques, the present study could provide the basis for understanding how proprioception is processed in the sensorimotor cortex.

Comparisons of the SEP distributions between anaesthetized macaques and behaving macaques highlight some potential limitations in the breadth of study over which our findings may be applicable. Previously, we described SR-evoked SEPs in SI of behaving macaques and also found small, long-latency SEPs in MI and the premotor cortex [54]. In the present study, using anaesthetized macaques, the latter was found in one monkey (SO) but not in the other (TA). It seems likely that the level of anaesthesia preferentially affected multi-synaptic activation in MI neurons, and resulted in inconsistent results in the two monkeys. The difference could also be supported by the fact that SEPs in MI and premotor cortex were more strongly affected by behavioral context [54], suggesting a high sensitivity of MI SEPs to the overall cortical state.

## Conclusions

We systematically examined the SEPs from muscle–joint and cutaneous nerves of the forearm (DR and SR, respectively) in SI of individual macaques. DR stimulation evoked focal activation at the bottom of the posterior part of CS in area 3a, whereas SR stimulation evoked widespread activation in SI, with large-amplitude and short-latency SEPs clustered in the superficial part of the posterior part of CS. Our results suggest that area 3a may not only convey muscle-afferent signals early in somatosensory processing but also integrates them with cutaneous afferent signals to shape proprioception.
